# Plasma Extracellular Vesicles as Liquid Biopsies for Glioblastoma: Biomarkers, Subpopulation Enrichment, and Clinical Translation

**DOI:** 10.3390/ijms262311686

**Published:** 2025-12-02

**Authors:** Abudumijiti Aibaidula, Ali Gharibi Loron, Samantha M. Bouchal, Megan M. J. Bauman, Hyo Bin You, Fabrice Lucien, Ian F. Parney

**Affiliations:** 1 Department of Molecular Pharmacology and Experimental Therapeutics, Mayo Clinic Graduate School of Biomedical Sciences, Mayo Clinic, Rochester, MN 55901, USA; aibaidula.abudumijiti@mayo.edu (A.A.); bouchal.samantha@mayo.edu (S.M.B.); 2 Department of Neurological Surgery, Mayo Clinic, Rochester, MN 55901, USA; gharibiloron.ali@mayo.edu (A.G.L.); bauman.megan@mayo.edu (M.M.J.B.); you.hyobin@mayo.edu (H.B.Y.); 3 Mayo Clinic Aix School of Medicine, Mayo Clinic, Rochester, MN 55901, USA; 4 Department of Immunology, Mayo Clinic, Rochester, MN 55901, USA; lucien-matteoni.fabrice@mayo.edu; 5 Department of Urology, Mayo Clinic, Rochester, MN 55901, USA

**Keywords:** extracellular vesicles, glioblastoma, liquid biopsy, plasma biomarkers

## Abstract

Glioblastoma (GBM), the most common primary malignant brain tumor in adults, has a median survival of 14–15 months despite aggressive treatment. Monitoring relies on MRI, but differentiating tumor progression from pseudo-progression or radiation necrosis remains difficult. Plasma extracellular vesicles (EVs) are emerging as promising non-invasive biomarkers due to their molecular cargos and accessibility. This review evaluates studies that specifically isolated plasma EVs for molecular profiling in GBM diagnosis and monitoring. Biomarkers (miRNA, RNA, DNA, proteins), EV characterization methods, and advancements in enriching tumor-derived EV subpopulations and assessing their diagnostic and prognostic potential are highlighted. Plasma EVs carry diverse cargos, including miRNAs (e.g., miR-21, miR-15b-3p), mRNAs (e.g., EGFRvIII), circRNAs, and proteins (e.g., CD44, GFAP). Composite molecular signatures have achieved sensitivities of 87–100% and specificities of 73–100% for GBM diagnosis. Tumor-derived EVs, enriched using techniques like SEC-CD44 immunoprecipitation, microfluidic platforms, or 5-ALA-induced PpIX fluorescence, enhance biomarker detection. Non-tumor-derived EVs may also reflect GBM’s systemic effects. Challenges include EV heterogeneity, non-EV contamination, and variable biomarker expression across studies. Plasma-EV-based liquid biopsies offer significant potential for GBM monitoring, with advanced enrichment methods improving tumor-specific biomarker detection. Standardizing isolation protocols and validating biomarkers in larger cohorts are critical for clinical translation.

## 1. Introduction

Glioblastoma (GBM) is the most common primary malignant brain tumor in adults, with the average annual age-adjusted incidence rate (IR) of GBM at 3.19 per 100,000 population [1]. Standard treatment includes maximal surgical resection followed by radiation therapy and chemotherapy. Despite enormous efforts toward advancements in treatment, the median survival of patients with GBM remains just 14–15 months [2,3,4]. Patients are usually followed up clinically after surgery and at regular intervals thereafter with clinical evaluation and magnetic resonance imaging (MRI) scans to identify tumor progression or recurrence. However, MRI interpretation in the context of prior surgery and radiation therapy is challenging and subject to interpretive variability. In particular, pseudo-progression or radiation necrosis, which occur in a significant percentage of GBM patients, often exhibit similar radiographic features to true tumor progression, making accurate diagnosis and clinical decision-making challenging [5,6]. Biopsy surgery can provide a definitive diagnosis but also carries risks, including intracranial hemorrhage [7].

IDH and TERT promoter mutations, MGMT methylation, EGFR gene amplification, PTEN tumor suppressor gene deletion, ATRX mutation, and TP53 mutation, CDKN2A/B homozygous deletion mutation, as well as combined gain of entire chromosome 7 and loss of entire chromosome (+7/−10) are well-studied biomarkers for GBM [8,9]. However, they are mainly used for preoperative diagnosis, risk stratification, and treatment decisions. Currently, no established biomarkers for GBM disease monitoring are clinically available. Extracellular vesicles (EVs) are promising biomarkers for glioblastoma disease monitoring due to their abundance in biofluids, easy accessibility, and rich molecular cargos that are reflective of their parent cells [10,11,12,13]. Extracellular vesicles are lipid bilayer nanoparticles and can be classified into exosomes, microvesicles, and large oncosomes based on their size and cell of origin [14]. They can be found in various biofluids, including saliva, urine, cerebrospinal fluid, and plasma [15]. Extracellular vesicles found in plasma originate from cells that interact with the blood flow. This includes GBM tumor cells due to the destruction of the blood–brain barrier [16,17,18]. This presents an opportunity to utilize plasma extracellular vesicles as a non-invasive diagnostic tool for monitoring GBM disease, enabling molecular assessment of treatment response and early detection of recurrence or progression, thereby complementing neuroimaging and enhancing surveillance precision.

Plasma extracellular vesicles are more easily obtained than cerebrospinal fluid (CSF) extracellular vesicles, thus making them the most widely studied source of extracellular vesicles. Liquid biopsy studies employ different methods to identify molecular profiles from plasma samples. For instance, miRNA signatures can be derived directly from total plasma without EV isolation [19,20,21,22,23] or, alternatively, from separated and concentrated total plasma EVs [20,24] or specifically enriched EV subpopulations [25,26]. These methodological differences may contribute to the variability in significant miRNAs reported across studies [19,21,22,23,24]. Given that EVs are more enriched in genetic material compared to unfractionated plasma [27], molecular profiling of enriched plasma EV samples offers higher contrast, potentially enabling the identification of biomarkers with greater clinical relevance. Moreover, recent progress in EV sorting technology presents new opportunities to identify and enrich different plasma EV subpopulations, particularly those originating from tumor cells [26,28,29]. Tracing biomarkers from tumor-derived EVs could help develop more specific biomarkers and improve the accuracy of GBM liquid biopsy.

Here, we present a comprehensive review of current GBM plasma liquid biopsy studies, focusing on those that specifically outline methods for plasma EV separation and concentration. Additionally, by summarizing specific markers used to characterize and quantify plasma EVs, and recent advances in GBM plasma EV subpopulation enrichment and molecular profiling, we highlight the promising future of plasma EV fractionation and provide a fresh perspective on the current GBM plasma EV biomarker research. The review is organized into three sections: (I) EV-based approaches for GBM diagnosis and monitoring, (II) strategies for plasma EV isolation and characterization, and (III) emerging techniques for enriching EV subpopulations to improve downstream analyses.

## 2. Part I—Extracellular-Vesicle-Based Liquid Biopsy for GBM Diagnosis and Monitoring

**Figure 1:**
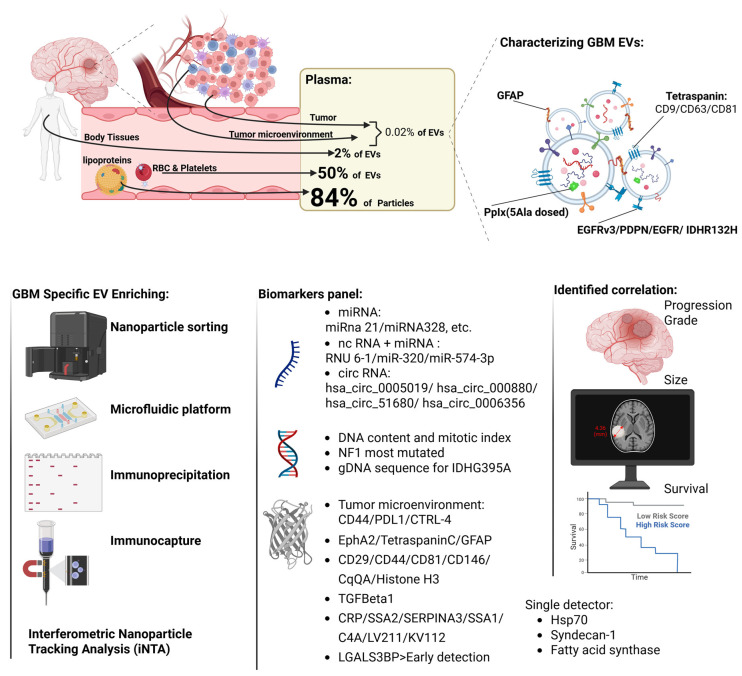
Extracellular vesicle (EV)-based strategies for biomarker characterization and clinical monitoring of glioblastoma (GBM) patients. Although circulating EVs are abundant, they are vastly outnumbered by lipoproteins, and most originate from platelets and erythrocytes rather than tissue or tumor sources; tumor-derived EVs therefore constitute only a minor subset of plasma EVs in GBM patients. Despite this rarity, multiple enrichment approaches, including immunocapture targeting GBM-specific proteins, lineage-specific immunoprecipitation, microfluidic technologies, and nanoparticle-based sorting, enable selective isolation of GBM-derived EVs. These strategies substantially enhance detection of tumor-derived signals, including RNA species, DNA mutations, tumor microenvironment–associated proteins and antigens, and individual molecular markers, with numerous studies demonstrating associations between enriched EV biomarkers and patient clinical variables.

GBM plasma EVs contain a diverse variety of intramembranous cargo, including DNA, mRNA, miRNA, and functional proteins (Table 1) [30,31]. This cargo represents multiple potential biomarkers, the measurement of which may serve to streamline GBM diagnosis and monitoring (Figure 1). miRNA-based EV cargo has been explored as a source of novel biomarkers in GBM. Tzaridis et al. found that compared with EVs from controls, EVs derived from GBM patients displayed significantly higher levels of miR-15b-3p, miR-21-3p, miR-155-5p, and let-7a-5p [25]. Furthermore, combining miRNA markers yielded prognostically significant subgroups, and the expression of miR-15b-3p, miR-21-3p, and miR-328-3p is negatively correlated with survival, whereas miR-106a-5p is positively correlated. MiR-21, although a well-known oncomiR that is upregulated in multiple malignancies and therefore not specific for GBM, was also identified as one of multiple potentially significant GBM EV cargo biomarkers by Akers et al., though the relative distribution of miRNA in plasma exosomes was difficult to predict [32]. Olioso et al. provided additional support for miR-21 as a candidate biomarker and found that increased expression of miR-21, miR-222, and miR-124-3p after resection was associated with the progression of high-grade glioma [33]. They also found that higher exosomal miRNA expression was correlated with lower progression-free survival and overall survival. Similarly, Ebrahimkhani et al. identified 26 miRNAs that were differentially expressed in GBM EVs compared to those from healthy donors, with seven miRNAs (miR-182-5p, miR-328-3p, miR-339-5p, miR-340-5p, miR-485-3p, miR-486-5p, and miR-543) considered most stable or predictive of GBM [24]. One study demonstrated that specific miRNAs (miR-9a-5p, miR-16-5p, miR-21-5p) were found in higher concentrations in patients with shorter survival [34]. Finally, Shao et al. identified miR-454-3p as a potential marker that is upregulated in GBM plasma EVs and associated with lower overall survival, which also decreased postoperatively [34]. Interestingly, aside from miR-21 and miR-328, there appears to be little overlap in candidate miRNA biomarkers between studies. It may thus be prudent to combine multiple miRNA candidates into a single “miRNA signature” to increase the utility and accuracy of liquid biopsy assays.

Importantly, other forms of RNA cargo in GBM-derived EVs may serve as useful diagnostic or prognostic indicators. Manterola et al. found that combining particular ncRNAs and miRNAs (RNU6-1, miR-320, and miR-574-3p) into a GBM EV signature was more predictive of a GBM diagnosis (sensitivity 87%, specificity 86%) than individual biomarkers alone (sensitivity and specificity from 59–73%) [20]. Another study that combined miRNAs and mRNAs identified 569 differentially expressed genes in GBM circulating EVs, achieving a biomarker panel for several types of glioblastomas with high sensitivity (89–100%) and specificity (73–100%) [37]. Furthermore, PCR-based GBM EV assays have been used to reliably detect EGFRvIII mRNA in patient samples with a specificity of up to 97.67% [38,39,40]. Additionally, Li et al. explored circRNAs as potential biomarkers and found that short-exon and suppressor circRNAs were more enriched in exosomes than in glioma cells. Specifically, they identified four subtypes (hsa_circ_0005019, hsa_circ_0000880, hsa_circ_0051680, and hsa_circ_0006365) that were associated with improved prognosis and could aid in glioma diagnosis [41].

DNA-based cargo has also been explored as a biomarker in GBM EVs, albeit in a more limited fashion. Piazza et al. found that exosomal DNA and tumor volume were linearly correlated in recurrent but not newly diagnosed GBM, and that EV DNA content and mitotic index were highly positively correlated in recurrent GBM [44]. They also found that the amount of exoDNA was inversely correlated with hypointense tumor volume in newly diagnosed GBM. Interestingly, Rosa et al. also found that GBM EVs isolated from patient plasma contained significantly less DNA than control EVs, with *NF1* being the most frequently identified and mutated gene in their cohort [45]. DNA-based assays thus produce distinct results for recurrent versus newly diagnosed GBMs, a vital consideration for developing a liquid biopsy based on such methods. Interestingly, using the gDNA sequence for IDH1^G395A^, Garcia-Romero et al. detected GBM EVs in patient blood samples regardless of blood–brain barrier permeability [17].

Proteomic signatures and protein cargo may be used to identify and quantify GBM EVs in patient samples [51,52]. As in RNA-based assays, proteomic signatures have the advantage of capturing multiple unique markers from heterogeneous tumors, an especially important consideration for GBM. For example, Osti et al. used proteomic data from patient plasma to construct an 11-component signature of GBM EV proteins [11]. Similarly, Dobra et al. defined a characteristic protein profile of 10 serum and 17 EV proteins for use in monitoring CNS tumors [56]. Using mass spectrometry of GBM EVs, Cilibrasi et al. defined an inflammatory biomarker signature and found that expression of this signature appears to be elevated in GBM EVs compared to those from healthy donors, indicating enrichment for biological processes mainly associated with complement activation, immune response, and B-cell activity [46]. Confirmed via cytology, sEVs from the plasma of glioma patients exhibited enrichment of ephrin type-A receptor 2 (14.7-fold), tenascin C (22.7-fold), and glial fibrillary acidic protein (8.4-fold) [62]. Glioblastoma patients exhibited significant upregulation of CD29, CD44, CD81, CD146, CqQA, and histone H3 compared to healthy volunteers, with additional upregulation of C1QA, CD44, and histone H3 observed to those with stable disease [48]. Cumba-Garcia et al. found that a group of immune markers, including IFN-γ, IL-10, B7-1, B7-2, ICOSL, and IL-3, is downregulated in GBM EVs compared to those from healthy donors [47]. TGF-β1 expression is also specific to GBM plasma EVs [53]. Muller-Haegele et al. demonstrated that exosomal protein levels were correlated with tumor grade [43]. Similarly, Hallal et al. clustered EV protein profiles according to histological subtype and grade; interestingly, repeat EV samples from patients with recurrent disease clustered with more aggressive glioma samples [49]. Rana et al. also found that proteins CRP, SAA2, SERPINA3, SAA1, C4A, LV211, and KV112 were differentially expressed in the three glioma subtypes (Grade I, II, and III) [50]. LGALS3BP, especially, is upregulated in EVs from glioma patients and has the potential for early glioma detection (sensitivity 77.8%, specificity 35.5%). As in RNA-based studies, the GBM EV proteomics literature exhibits little overlap, making the identification of a clear signature for clinical use challenging. Some groups have narrowed their analyses to a single protein biomarker, such as Hsp70 [55], syndecan-1 [54], or fatty acid synthase [57]; however, it is unlikely that a single biomarker will adequately capture the intratumoral heterogeneity of GBM, individual differences between patients, and variable responses to treatment.

A focused review of survival data shows that numerous EV-derived biomarkers have significant prognostic associations in GBM. Elevated levels of several EV components, including total EVs, key miRNAs (miR-15b-3p, miR-21-3p/5p, miR-328-3p, miR-454-3p), and EGFRvIII, are consistently linked to poorer survival, whereas markers such as miR-106a-5p and specific circRNAs are associated with more favorable outcomes. Collectively, these findings highlight the prognostic relevance of EV cargo in GBM (Table 2).

GBM-derived extracellular vesicles contain a broad array of nucleic acid and protein cargo with significant potential for noninvasive diagnosis, molecular characterization, and longitudinal disease monitoring. Across biomarker categories, composite EV-based signatures consistently demonstrate superior performance compared to single-analyte assays and may represent the most promising framework for developing clinically actionable liquid biopsy platforms. Importantly, distinctions between newly diagnosed and recurrent GBM should be accounted for when designing EV-based assays. Despite the growing number of proposed biomarkers, the limited concordance among studies continues to pose a major challenge to establishing a standardized clinical signature.

## 3. Part II—Identifying Tumor-Derived Extracellular Vesicles Among Non-Neoplastic EVs

Plasma EVs are very heterogeneous, reflecting their cells of origin. Currently, there is no established set of biomarkers for characterizing plasma EVs in GBM. Such vesicles are typically identified using either general EV markers or markers specific to GBM cancer cell lines (Table 3). EV tetraspanins are transmembrane proteins that serve many vital functions, some of which include signaling, cell adhesion, and membrane organization. These tetraspanins are shared by bloodborne EVs and tissue-derived EVs [65]. Multiple parameter detection of EV-associated tetraspanins—CD9, CD63, CD81—has been accomplished on single EVs using imaging flow cytometry [66]. Specifically, GBM patients have demonstrated increased double-positive CD63+/CD81+ and CD9+/CD63+ circulating EVs compared to normal controls [66]. However, given that these markers are not tumor-specific, it is impossible to differentiate whether the increased EV populations found in GBM patients are solely tumor-derived or secondary effects of the tumor. Instead, the detection of glial-associated markers (i.e., GFAP+) allows for more discrete localization of the EVs to the astrocytic cells of the central nervous system (CNS). Although GFAP is expressed in non-glial cells, GFAP positivity in EVs may be a valuable addition to a broader multimarker panel. In fact, increased GFAP+ EVs have been found in patients with malignant gliomas compared to healthy controls both before and after resection, suggesting that an elevated GFAP+ EV burden reflects the presence of underlying CNS pathology [67,68]. However, when EV subpopulations are followed longitudinally across the perioperative period, dynamic changes can provide prognostic information. For example, Sartori et al. observed that postoperative increase in GFAP+/TF+ EVs was associated with disease progression following tumor resection, indicating that persistent or rising levels of specific EV subsets may signal residual or recurrent disease [67]. This release of neuron-derived EVs into the periphery has been further amplified through blood–brain barrier (BBB) disruption via MR-guided focused ultrasound (FUS) to further enrich circulating biomarkers in liquid biopsy [69]. However, this GFAP+ EV strategy is still limited in its ability to differentiate tumor-derived EVs from non-tumor astrocytes. These observations are consistent with EV studies performed on glioma tissue, where bulk EV preparations inevitably contain vesicles released both by malignant glioma cells and by non-neoplastic astrocytes and other stromal cells in the tumor microenvironment. Rather than representing a technical artifact, this mixed cellular origin is biologically relevant, as astrocyte-derived EVs can modulate tumor growth, invasion and treatment resistance, and some ‘tumor-associated’ signatures in plasma EVs likely reflect contributions from these reactive astrocytes. “Tumor-derived” and “non-neoplastic” EVs should therefore be interpreted along a continuum of EVs originating from both neoplastic cells and the surrounding microenvironment, rather than as completely separable populations.

Alternatively, Shao et al. found that microvesicles and EVs derived from GBM in vitro cell lines and patient plasma have unique protein signatures that include EGFR, EGFRvIII, PDPN, and IDH1 R132H as potential GBM-associated biomarkers [71]. In this study, determining expression levels of EGFR, EGFRvIII, PDPN, and IDH1 R132H together allowed for the distinction of GBM versus normal donor EVs. Similarly, Manda et al. found that exosomal expression of the EGFRvIII transcript was present in 43 of 96 patients’ serum (44.79%) and correlated with poor patient survival [64]. For applications within liquid biopsy, the ddPCR assay has enabled a sensitivity of 73% and specificity of 98% in detecting EGFRvIII mutation in plasma EVs [39]. Likewise, the use of this biomarker as a target for microfluidic isolation can result in simultaneous detection of EGFRvIII-containing EVs at 94% tumor-EV specificity and tumor-RNA enrichment, allowing for downstream analysis without any additional isolation steps [38]. Unfortunately, biomarkers such as EGFRvIII and IDH1 R132H are not present in all GBM tumors, which limits the applicability of single biomarker-based detection and enrichment methods.

Due to alterations in hemoglobin metabolism, GBM cells rapidly uptake 5-aminolevulinic acid (5-ALA), which is metabolized into fluorescent protoporphyrin IX (PpIX) and accumulates within cells [72]. Preoperative administration of 5-ALA and the resulting fluorescence of PpIX has allowed for increased visualization of GBM tumor at surgery. Similarly, Jones et al. found that glioma cells dosed with 5-ALA release 247-fold higher PpIX+ EVs compared to sham-dosed glioma cells. Additionally, GBM patient plasma following 5-ALA administration was found to contain significantly higher levels of circulating PpIX+ EVs than the pre-dosing baseline. In these cases, the rise in PpIX+ EV signal was correlated with increased radiographic tumor volumes of enhancement [59]. These results were also reinforced by Maas et al., who demonstrated that 5-ALA administration leads to the accumulation of fluorescent PpIX in patient-derived and cell-cultured EVs, which were detected using high-resolution flow cytometry [60]. Additionally, the authors further characterized EVs using ddPCR, which indicated that PpIX+ EVs contained glioblastoma-associated miRNAs, thereby validating the tumor origin of PpIX+ EVs.

Though all the EV markers above could be analyzed by conjugating additional fluorophore followed by flow cytometry, due to the detection sensitivity of different flow cytometry platforms, the target EV concentration varies significantly between studies, and comparative studies are not feasible due to the lack of calibrated instruments in most EV studies [73]. Many studies have only used qualitative analysis and have not determined a threshold for findings of increased levels of potential biomarkers. It remains unclear whether these results indicate an increase in target EV populations specific to GBM or simply reflect the disease state. For tumor-associated biomarkers like EGFR/EGFRvIII/IDH, the dynamic changes in these biomarkers throughout the whole disease status should be studied; thus, fold change compared to baseline could be individualized in each patient. Finally, most published studies are either descriptive or comparative analyses in relatively small cohorts. Expanding those established analytical pipelines could not only further demonstrate variations among different patients but also show the correlation between tumor volume and patient survival. Most studies lacked a validation set, thereby precluding the assessment of the data’s sensitivity and specificity.

## 4. Part III—Characterizing and Enriching Plasma EV Subpopulations Can Improve Downstream Plasma EV Analysis

Circulating EVs are present with numerous other non-EV particles of similar size in plasma. Traditional plasma EV isolation protocols like ultracentrifugation and size exclusion chromatography (SEC) do not completely separate EVs from lipoproteins and protein aggregates with similar size and density, which are more abundant than EVs [74,75]. Common isolation workflows lead to impure EV preparations that can negatively affect EV surface integrity, RNA recovery, and protein purity [76]. Enriching plasma EVs could elucidate the source of miRNAs, since some evidence supports that circulating miRNAs are bound to the Ago2 ribonucleoprotein complex [77] and can be co-isolated with plasma EVs, while some suggest that the reported miRNAs originate from circulating EVs [27,78]. The inconsistent regulatory status of reported miRNAs in GBM plasma may be due to the co-isolation of non-EV particles with plasma EVs. miR-185 was reported as downregulated in the GBM total plasma [21] but was upregulated in isolated GBM plasma EVs compared to normal donors [24]. miR-15p also has conflicting results among different studies [19,22,79]. CD44 is highly expressed in glioblastoma mesenchymal stem cells (GBMCs), where it is associated with a mesenchymal phenotype and enhanced tumor aggressiveness. Through its interaction with hyaluronic acid (HA), CD44 mediates cell migration, adhesion, and intracellular signaling pathways that support glioma growth and survival [80]. Tzaridis et al. applied a combination of SEC and CD44 immunoprecipitation to isolate GBM plasma EVs. They found that this significantly decreased the yield of contaminating calnexin and lipoproteins in plasma EV samples [25]. By comparing the expression profiles of 8 well-reported miRNAs between SEC-CD44+ EVs and total serum, they found that five of them were enriched in SEC-CD44+ EVs, while two were enriched in total serum. Sabaté Del Río et al. developed a lab-on-a-chip device that utilizes dielectrophoretic (DEP) separation principles to remove lipoproteins, which could provide a novel mechanism for enriching EVs [81]. Currently, for quantification purposes, interferometric nanoparticle tracking analysis (iNTA) studies the refractive index to identify nanoparticles with high sensitivity and precision [82]. Similarly, a quantitative sandwich immunoassay for CD63 and EGFR using charge-gating with a hydrophilic anion exchange membrane and charged silica nanoparticle reporter functionalized with capture and detection antibodies yielded an AUC of 0.99 (*p*-value < 0.001) [83]. Ultimately, more specific enrichment of plasma EVs is appealing as EVs have a higher concentration of miRNAs compared to total serum, and a subset of miRNAs is not detected in unfractionated serum [27].

The heterogeneity of plasma EVs is further compounded by the fact that they represent various cell types in the bloodstream. Characterizing and enriching plasma EVs can selectively identify and enrich target EV subpopulations (Table 4). Blood-borne EVs are the most abundant EVs in circulation; however, lipoproteins outnumber EVs in plasma by six orders of magnitude [84]. It is estimated that more than 50% of plasma EVs may come from platelets and erythrocytes [16]. Tissue- and tumor-derived EVs constitute a minor subset of circulating plasma EVs. Transcriptomic analyses indicate that approximately 0.2% of plasma EVs originate from tissue sources [85], whereas proteomic profiling suggests that only 0.02–0.05% of EV-associated proteins in plasma are of tumor origin [86]. Tumor-derived EVs are rare in GBM patient plasma (<10%), but using an immunocapture method (biotinylated EVS captured by streptavidin-coated coverslip targeting EGFR, EGFRvIII, EpCAM, and IDH1-R132H positive EVs), Fraser et al. found that they could enrich those rare tumor-derived EVs and analyze their surface marker expression profiles [58]. Immunoprecipitation targeting the cellular origin of glioma, including astrocytes (GLAST and EAAT2), oligodendrocyte precursor cells (OSP and MOG), and neural stem cells (CD133), could isolate glioma-specific sEVs [62]. By utilizing a microfluidic platform (EVHB-Chip) with antibodies against EGFR, EGFRvIII, ephA2, podoplanin, PDGFR, and MCAM, Reategui et al. also achieved a 10-fold increase in GBM plasma tumor EV concentration with less background RNA from non-tumor EVs [87]. Recent progress in nanoparticle sorting also empowers plasma EV enrichment [29]. Hsia et al. sorted PpIX+ EVs from GBM patient plasma and found that this significantly improved the detection of tumor-derived genes [26]. They also reported that only 50.6% of the genes commonly identified in both PpIX+ EVs and tumor tissue are expressed in the total plasma EVs.

Even EVs released from the same parent cells could vary widely with respect to their cell surface marker expression as well as their cytosolic components [89]. Fraser et al. also examined the putative EV markers (integrin beta 1, CD9, CD63, and CD81) and glioblastoma markers (EGFR, EGFRvIII, EPCAM) on EVs isolated from GBM tumor cell cultures [58]. They found that only part of EVs expressed those markers, with the highest expressed putative EV marker being integrin beta 1 (52%) and the tumor marker being EGFR (32%). PpIX is a specific marker for GBM EVs [59,60]. This variability was also observed in GBM patient plasma EVs [58]. Smith et al. also found that single exosomes isolated from the same cells exhibited high spectral variability in reading their total exosomal components [90]. There may be multiple methods available to identify and characterize these subpopulations, enabling performance to stratified analyses. For example, Su et al. demonstrated a charge-based fractionation method that effectively isolates and fractionates EV subpopulations based on surface charge [91]. A more comprehensive analysis of the plasma EV subpopulations, classified based on a single marker, may uncover more specific and consistent insights into their molecular signatures.

To date, efforts to discover plasma EV biomarkers are underway with the aim of identifying specific markers, such as IDH, GFAP, and EGFRvIII, that were previously identified in glioma tissue cells within these vesicles [13,30]. This can be highly specific but has low sensitivity and is technically challenging. Not all tumor cells express the same tumor marker in a positive tumor [92]. Tumor-derived EVs are rare in GBM plasma, and they vary in their tumor marker expression [58]. Tumor EV concentrations and their EV tumor marker expressions also vary between patients [58]. This raises the intriguing hypothesis that those already identified cancer-associated molecular signatures may originate from non-tumor-derived EVs—including those released by astrocytes and other tumor-associated stromal cells—further supporting the compelling hypothesis that GBM is a systemic disease, though they never metastasize outside of the CNS [93,94,95]. CD44 is expressed on multiple immune and glial cell populations, including B and T lymphocytes and astrocytes [96], and is highly upregulated in GBM serum EVs [25,97]. As a result, CD44-positive EVs likely include vesicles released both from glioma stem-like cells and from tumor-associated astrocytes and other stromal cells within the glioma microenvironment. Notably, CD44+ EVs carry GBM-associated miRNAs that enable prognostic stratification, underscoring how microenvironment-derived EVs can contribute to clinically useful “tumor-associated” liquid-biopsy signatures. CD9, one of the classical EV markers, is the highest expressed tetraspanin on the surface of GBM patient plasma EVs, and it is overexpressed compared to healthy subjects [66]. Replicated in a GBM microenvironment in vitro, EVs from tumor explants demonstrated an increase in multiple potential markers that putatively originate from non-CNS cells, such as CD146 (MCAM) [89]. Ishwar et al. also found that EVs from NK cells in the plasma carry unique tumor-specific signals marked by increased PD-L1 and CTRL-4 expression [98]. This is well supported by the concept that immune cells could effectively cross the BBB via transcellular cytosis, disrupted BBB, or gap junctions [99]. Those emerging studies shed light on the possibilities of developing non-tumor-derived EVs as a source of GBM biomarker discovery.

Plasma EVs circulate with other non-EV particles and are heterogeneous in terms of their cell of origin. Current EV isolation techniques could enrich plasma EVs, but cannot completely separate plasma EVs from non-EV particles. Characterizing plasma particles based on specific marker expression could differentiate EVs from non-EVs, better group plasma EVs into specific subpopulations, and accurately identify the cells of origin. Coupled with recently available techniques such as nanoparticle sorting, future research could reveal biomarkers that are unique to each subpopulation, thereby enabling the development of a biomarker panel with high accuracy and potentially creating a clinical tool.

An additional limitation of the current literature is the limited attention to control group selection. Most of the studies summarized here contrast glioma or GBM cohorts with “healthy” donors, often volunteers or patients undergoing routine phlebotomy, with minimal reporting of comorbidities. Common chronic disorders, such as benign tumors (e.g., prostate hyperplasia, uterine fibroids), liver disease, allergic or autoimmune processes, can all contribute EVs from non-cerebral inflammatory or neoplastic sites. Without careful phenotyping and matching of these donors, such background EV signals may confound glioma-associated signatures and limit reproducibility across cohorts.

For certain clinical questions, neurosurgical “disease controls” may therefore be more informative than broadly defined healthy donors. Patients undergoing surgery for non-glial intracranial lesions such as meningiomas or cerebral vascular malformations present with similar symptoms and radiographic findings but lack infiltrative glioma biology. These cases also provide a unique opportunity to analyze EVs obtained directly from resected tissue alongside matched pre- and postoperative plasma samples. Systematic comparison of tissue-derived and plasma EV profiles between these disease controls and glioma patients could clarify the relative contribution of tumor-derived versus non-neoplastic EVs and refine the specificity of proposed plasma EV biomarkers.

## 5. Conclusions

Plasma extracellular vesicles are an attractive source of biomarkers for liquid biopsy in glioblastoma patients; however, currently available techniques for their enrichment and analysis are highly variable and require additional validation. New approaches for differentiating GBM-derived from non-neoplastic plasma EVs and enriching plasma EV subsets are promising methods to improve downstream biomarker analysis. Although EVs are not yet recommended for routine clinical use due to the absence of fully validated, standardized, and high-throughput commercial assays required for clinical decision-making, they hold substantial translational promise. EVs can traverse the blood–brain barrier and provide noninvasive molecular information (e.g., EGFRvIII, IDH mutation status) relevant for diagnosis, prognostication, and monitoring treatment resistance. Participation in clinical trials and research initiatives that incorporate EV analyses is strongly encouraged, with strict adherence to standardized sample collection and processing protocols [100] to improve data rigor and reproducibility.

## Figures and Tables

**Table 1:** Biomarkers identified in GBM plasma extracellular vesicles. Please refer to the reference column in the table for more information. AA, Anaplastic Astrocytoma; Alix1, Apoptosis-linked gene-2-interacting protein 1; ARF6, ADP-ribosylation factor 6; AUROC, Area Under the Receiver Operating Characteristic curve; BBB, Blood–Brain Barrier; CD, Cluster of Differentiation; CFDA, Carboxyfluorescein Diacetate; EGFR, Epidermal Growth Factor Receptor; EGFRwt, Epidermal Growth Factor Receptor wild type; EGFRvIII, Epidermal Growth Factor Receptor variant III; EpCAM, Epithelial Cell Adhesion Molecule; EV, Extracellular Vesicle; GBM, Glioblastoma; gDNA, genomic DNA; HSP70, Heat Shock Protein 70; LGG, Low-Grade Glioma; NanoFACS, Nanoparticle Fluorescence-Activated Cell Sorting; NR, Not Reported; NSCLC, Non–Small Cell Lung Cancer; (*n*), Number; PpIX, Protoporphyrin IX; TGF-β1, Transforming Growth Factor beta 1; TMZ, Temozolomide; TSG101, Tumor Susceptibility Gene 101; VAMP3, Vesicle-Associated Membrane Protein 3.

Biomarker	EV Isolation Method	Case (*n*)	Control (*n*)	Sensitivity	Specificity	References
miR-21, miR-222 and miR-124-3p	ExoQuick-TM	GBM (55); AA (5)	Patients served as their own internal controls	ROCAUC: 0.93 miR-21; 0.84 miR-222; and 0.88 miR-124-3p		Olioso, D. et al., 2021 [33]
miR-454-3p	Ribo Exosome Isolation Reagent	Glioma (24)	Healthy donors (24)	79.17%	91.67%	Shao, N. et al., 2019 [34]
miR-21, miR-103, miR-24, and miR-125	differential ultracentrifugation	GBM (9)	None	NR	NR	Akers, J. et al., 2015 [32]
let-7i, miR93 and miR-151a	differential ultracentrifugation	GBM (15)	GBM (No TMZ exposure)	NR	NR	Zeng, A. et al., 2018 [35]
miRNA profile	size-exclusion chromatography	GBM (55)	Healthy donors (10)	NR	NR	Tzaridis, T. et al., 2020 [25]
miRNA profile	Size exclusion chromatography	GBM (16, IDH wt), Grade II-III (10, IDH mut)	Healthy donors (25), non-glioma (10)	AUROC 0.84		Ebrahimkhani, S. et al., 2018 [24]
miRNA profile	modified ExoQuick with immunoprecipitation	GBM (31, Grade 3 and 4 GBM, grade 2 and 3 astrocytoma)	Healthy donors (9)	NR	NR	Deep et al., 2024 [36]
miRNA and non-coding RNA	ExoQuick precipitation solution	GBM (25 study set, 50 validation set)	Healthy donors (25)	87% (cutoff value of 0.349 for the 3 sncRNAs)	86% (cutoff value of 0.349 for the 3 sncRNAs)	Manterola, L. et al., 2014 [20]
miRNA and mRNA	ExoRNeasy Serum/Plasma Midi Kit	GBM (91)	Healthy donors (31)	89–100%	73–100%	Mut et al., 2023 [37]
EGFRvIII mRNA	microfluidic isolation	GBM (13)	Healthy donors (6)	NR	94% tumor-EV specificity	Reátegui, E. et al., 2018 [38]
EGFRvIII mRNA	ExoRNeasy Maxi Kit	GBM (30, EGFRvIII)	GBM (10, EGFRwt), healthy donors (14)	72.77%	97.67%	Batool, S. et al., 2022 [39]
EGFRvIII mRNA	differential ultracentrifugation	GBM (30)	Healthy donors (30)	7/25 detected EGFRvIII	NR	Skog, J. et al., 2008 [40]
circular RNA	Total Exosome Isolation	High grade astrocytoma (30)	Healthy donors (12)	hsa_circ_0075828 (96.67%) hsa_circ_0002976 (93.33%) hsa_circ_0003828 (73.33%)	hsa_circ_0075828 (99.92%) hsa_circ_0002976 (91.67%) hsa_circ_0003828 (83.33%)	Li, P. et al., 2022 [41]
Exosomal mRNA (PTEN, YAP1, LOX)	total Exosome Isolation Kit	Glioma (106, Grade IV 62, Grade III 14, Grade II 26, Grade I 4)	Healthy donors (20)	71.6% (PTEN)	NR	Patnam, S. et al., 2022 [42]
Total protein level and mRNA expression levels for 24 genes	size exclusion columns followed by differential ultracentrifugation	GBM (13), AA (5), AO (3), AOA (1)	Healthy donors (10)	NR	NR	Muller, L. et al., 2015 [43]
Concentration of exosomal DNA	membrane-based affinity	GBM (10), Grade III (1), Grade II (3 IDH mut, 2 IDH wt)	Other neurologic diseases (10)	NR	NR	Piazza, A. et al., 2022 [44]
IDH1^G395A^ gDNA sequence	differential ultracentrifugation, fast Cold PCR	Glioma (20), brain metastasis (1)	Intact BBB (3), disrupted BBB (18). No healthy donors.	NR	NR	Garcia-Romero et al., 2017 [17]
exosomal DNA (ATRX, CDKN2A, H3F3A, IDH1, IDH2, NF1, PTEN, TERT, and TP53)	differential ultracentrifugation followed by exoEasy maxi kit	GBM (10)	Healthy donors (5)	NR	NR	Rosa, P. 2022 [45]
Inflammatory biomarker signature	differential ultracentrifugation	GBM (15)	Healthy donors (10)	NR	NR	Cilibrasi, C. et al., 2022 [46]
Cytokine and checkpoint molecule arrays	density gradient ultracentrifugation	GBM (19)	Healthy donors (19)	NR	NR	Cumba Garcia, L. et al., 2019 [47]
Proteomic signature	modified ExoQuick with immunoprecipitation	GBM (31, Grade 3 and 4 GBM, grade 2 and 3 astrocytoma)	Healthy donors (9)	NR	NR	Deep et al., 2024 [36]
Proteomic signature	Exo-spin exosome purification kit	GBM (67, IDH wt)	Healthy donors (22)	AUROC 0.76		Tzaridis et al., 2023 [48]
Proteomic signature	size-exclusion chromatography	GBM (24 IDH wt, 2 IDH mut), Glioma Grade II–III (13 astrocytoma IDH mut; 4 oligodendroglioma IDH mut, 1p19q codeleted)	Meningioma (5, garde I), Healthy donors (6)	NR	NR	Hallal, S. et al., 2020 [49]
Proteomic signature	differential ultracentrifugation	Glioma (9, Grade I, II, or III, 3 in each group)	Healthy donors (3)	77.8% (galectin-3 BP)	35.5% (galectin-3 BP)	Rana, R. et al., 2021 [50]
Proteomic signature	differential ultracentrifugation	GBM (6)	Healthy donors (6)	NR	NR	André-Grégoire, G. et al., 2018 [51]
Raman spectra of plasma EVs	differential ultracentrifugation	GBM (46), brain metastasis (28), meningioma (28)	Lumbar disc herniation (36)	80–95%	80–90%	Bukva, M. et al., 2021 [52]
TGF-beta1	differential ultracentrifugation	High grade glioma (12)	Healthy donors (12)	NR	NR	Graner, M. et al., 2009 [53]
Syndecan-1	size exclusion chromatography	GBM (69), LGG (17)	Healthy donors (3)	79%	91%	Chandran, V.I. et al., 2019 [54]
HSP70	differential ultracentrifugation	GBM (34), NSCLC (166)	Healthy donors (108)	91%	33%	Werner, C. et al., 2021 [55]
Protein marker panel (17 EV proteins and 10 whole serum proteins)	differential ultracentrifugation	GBM (24), brain metastasis (24), meningioma (24)	Healthy donors (24)	NR	NR	Dobra, G. et al., 2020 [56]
Fatty acid synthase (FASN)	differential ultracentrifugation	GBM (18)	Healthy donors (12)	NR	NR	Ricklefs et al., 2020 [57]
Frequency of CD9, CD63, CD81, TSG101, Alix, CD40, Arf6, VAMP-3, IDH1-WT, Integrin beta 1, EGFR, EGFRvIII, IDH1-R132H, EPCAM	differential ultracentrifugation	GBM (8)	Healthy donors (2)	NR	NR	Fraser, K. et al., 2019 [58]
Transcriptome profiles of EV subpopulations marked by PpIX, CD63, EFGR, CFDA	NanoFACS	GBM (8)	Healthy donors (2)	NR	NR	Hsia, T. et al., 2022 [26]
PpIX+ Evs	membrane-based affinity	GBM (6)	Same patients used as pre-dosing controls	NR	NR	Jones, P. et al., 2019 [59]
PpIX+ Evs	differential ultracentrifugation	GBM (30)	Same patients used as pre-dosing controls	NR	NR	Maas, S. et al., 2020 [60]
EV concentration and proteomic signature	differential ultracentrifugation	GBM (43)	Healthy donors (33), other CNS malignancies (25)	NR	NR	Osti et al., 2019 [11]
Quantity of microvesicles	differential ultracentrifugation	GBM (11)	Healthy donors (7)	NR	NR	Koch, C. et al., 2014 [61]

**Table 2:** EV-derived biomarkers in patients plasma reported to correlate with survival in GBM, Biomarkers for which higher levels are associated with reduced survival or improved survival are indicated, along with the corresponding reference for each study.

Biomarker	Survival	Reference
Total EVs in plasma	Higher levels associated with reduced survival	Ricklefs, F.L., et al 2024 [63]
miR-15b-3p, miR-21-3p, and miR-328-3p	Higher levels associated with reduced survival	Tzaridis et al., 2020 [25]
miR-106a-5p	Higher levels associated with increased survival	Tzaridis et al., 2020 [25]
Exosomal miRNA expression	Higher levels associated with reduced survival	Olioso et al., 2021 [33]
miR-9a-5p, miR-16-5p, miR-21-5p	Higher levels associated with reduced survival	Shao et al., 2019 [34]
miR-454-3p	Higher levels associated with reduced survival	Shao et al., 2019 [34]
EGFRvIII	Higher levels associated with reduced survival	Manda et al., 2018 [64]
hsa_circ_0005019, hsa_circ_0000880, hsa_circ_0051680, and hsa_circ_0006365	Higher levels associated with increased survival	Li et al., 2022 [41]

**Table 3:** Markers characterizing extracellular vesicles in GBM patient plasma. Please refer to the reference column in the table for more information. AA, anaplastic astrocytoma; AV, annexin V; CD, cluster of differentiation; EGFR, Epidermal Growth Factor Receptor; EGFRvIII, Epidermal Growth Factor Receptor variant III; EV, extracellular vesicle; GBM, glioblastoma; GFAP, glial fibrillary acidic protein; HHG, high-grade glioma; (*n*), number of patients/samples; NR, not reported; PDPN, podoplanin; TF, tissue factor.

Plasma EV Markers	EV Isolation Method	EV Concentration	EV Analysis Method	Case (*n*)	Control (*n*)	Sensitivity	Specificity	References
CD9, GFAP, survivin	differential ultracentrifugation	3.68 × 10^9^ particles/mL (among CD9+ EVs, 22.8% GFAP+; 9.1% SVN+; 6.8% GFAP+/SVN+)	ImageStreamX Mark II Imaging Flow Cytometer	GBM (8)	Healthy donors (3)	NR	NR	Galbo, P., Jr. et al., 2017 [68]
GFAP, CD62E, AV, TF	platelet-free plasma; no further processing	(Approximations) CD62E+/AV− = 3000 MPs/mL; AV+/CD62E− = 3000 MPs/mL; CD62E+/AV+ = 700 MPs/mL; TF+/GFAP− = 200 MPs/mL; GFAP+/TF− = 200 MPs/mL; GFAP+/TF+ = 300 MPs/mL	Cytomics FC500, Beckman Coulter Flow cytometry	GBM (41)	Healthy donors (20)	NR	NR	Sartori, M. et al., 2013 [67]
CD9, CD63, CD81	differential ultracentrifugation	(Median) GBM: CD81+ = 10^6^ EVs/mL plasma; CD9+ = 10^7^ EVs/mL plasma; CD63+ = 10^5^–10^6^ EVs/mL plasma. AA: CD81+ = 10^6^ EVs/mL plasma; CD9+ = 10^7^ EVs/mL plasma; CD63+ = 10^6^ EVs/mL plasma	Imaging flow cytometry and quantitative PCR (qPCR)	GBM (22), AA (7)	Healthy donors (19)	NR	NR	Ricklefs, F. et al., 2019 [66]
Annexin V, CD41, anti-EGFR, CD235	differential ultracentrifugation	Median: phosphatidyl-serine EVs = 1.08 × 10^3^; platelet EVs = 0.77 × 10^3^; EGFR EVs = 0.54 × 10^3^; RBC EVs= 0.67 × 10^3^	FACS-Canto Flow cytometry	GBM (16)	None	NR	NR	Evans, S. et al., 2016 [70]
EGFR, EGFRvIII, PDPN and IDH1 R132H	differential ultracentrifugation	NR	fluorescence reader, micronuclear magnetic resonance (μNMR) detection with microfluidic chip	GBM (24)	Healthy donors (8)	>90% accuracy	>90% accuracy	Shao, H. et al., 2012 [71]
CD56, CD171 (Neuron-derived EVs)	platelet-free plasma; no further processing	1000–10,000 particles/mL	nanoscale flow cytometry	GBM (9)	2 control patients with Alzheimer’s disease	NR	NR	Meng, Y. et al., 2021 [69]
EGFRvIII, CD81	total exosome isolation Kit	NR	BD FACSdiva flow cytometry	HGG (96)	Healthy donors (50), non-glioma (15)	81.58% (95% CI 65.67–92.26%)	79.31% (95% CI 66.65–88.83%)	Manda, S. et al., 2018 [64]
fatty acid synthase (FASN)	differential ultracentrifugation	Mean: FASN+ EVs 2.2 × 10^6^/mL; FASN+/CD63+ EVs 4.1 × 10^5^/mL; FASN+/CD81+ EVs 8.0 × 10^5^/mL	Imaging flow cytometry	GBM (18)	Healthy donors (12)	NR	NR	Ricklefs, F. et al., 2020 [57]

**Table 4:** Current strategies for separating and concentrating GBM plasma extracellular vesicles subpopulations. Please refer to the reference column in the table for more information. CD, Cluster of Differentiation; EGFR, Epidermal Growth Factor Receptor; EGFRvIII, Epidermal Growth Factor Receptor variant III; EphA2, Ephrin type-A receptor 2; EpCAM, Epithelial Cell Adhesion Molecule; EV, Extracellular Vesicle; GBM, Glioblastoma; MCAM, Melanoma Cell Adhesion Molecule; PDGFR, Platelet-Derived Growth Factor Receptor; TSG101, Tumor Susceptibility Gene 101; TMV, Tumor Microvesicle.

Samples	Enrichment Method	Downstream Analysis	Significant Findings	Reference
EVs collected from the U87-EGFRvIII cells spiked into plasma samples	Alternating current electrokinetic (ACE) microarray chip device	RT-RNA analysis	This is a feasibility study showing this method could isolate tumor-derived EGFRvIII EVs from whole blood and plasma samples This also enabled further detection of both their protein cargos (CD63 and TSG101) and specific mRNA biomarkers for mutated EGFRvIII in enriched EV samples	Ibsen et al., 2017 [88]
Plasma samples from GBM patients (*n* = 8) and healthy donors (*n* = 2)	Ultracentrifugation followed by biotinylated EVS captured by a streptavidin-coated coverslip Tumor EVs were labeled with EGFR, EGFRvIII, EpCAM, and IDH1-R132H	Image analysis for EV surface marker expression	Tumor EVs are rare (<10%) in GBM patient plasma Tumor markers expression varies among tumor-derived EVs The absolute TMV concentrations ranged considerably among subjects	Fraser et al., 2019 [58]
Plasma samples from GBM patients (*n* = 13) and healthy donors (*n* = 6)	Microfluidic platform (EVHB-Chip) with antibodies against EGFR, EGFRvIII, EphA2, podoplanin, PDGFR, and MCAM	Confocal microscopy and quantitative PCR.	This approach achieved a 10-fold increase in tumor EV enrichment Isolated plasma EVs have less background RNA from non-tumor EVs	Reategui et al., 2018 [38]
Serum from GBM patients (*n* = 55) and healthy donors (*n* = 10)	Size exclusion chromatography followed by CD44 immunoprecipitation	qRT-PCR analysis	Enriched plasma EV samples have decreased yield of calnexin and lipoproteins SEC + CD44 EVs have higher expression of miR-21-3p, miR-155-5p, miR-106a-5p, let-7a-5p and lower levels of miR-15b-3p and miR-23a-3p compared to unfractionated total serums	Tzaridis, T. et al., 2020 [25]
Plasma samples from GBM patients (*n* = 20) and healthy donors (*n* = 5), spiked DiFi cell-derived EVs into healthy human plasma	Charge-gating with a hydrophilic anion exchange membrane and charged silica nanoparticle reporter functionalized with capture and detection antibodies	Surface Plasmon Resonance (SPR), high-performance liquid chromatograph (HPLC), other orthogonal validation	The sensor demonstrated a limit of detection (LOD) of 30 EVs per μL, which is 1000× more sensitive than conventional ELISA-based methods. Achieved AUC of 0.99 and *p*-value of 0.000033 using EGFRvIII. Sensor was cross-validated with SPR, ELISA, nanoparticle tracking analysis (NTA), and differential ultracentrifugation	Maniya et al., 2023 [83]
GBM cell lines; plasma samples of GBM patients (*n* = 8) and healthy donors (*n* = 2)	Astrios NanoFacs Sorting (EV-PpIX, EV-CD63, EV-CD9, EV0-EGFR, EV-CFDA)	MiSeq sequencing	Different EV subpopulations have unique transcriptome profiles PpIX+ EVs have closer alignment to the tumorigenic process compared to other subpopulations lncRNA abundance and tumor-derived genes increased after enrichment	Hsia et al., 2022 [26]

## Data Availability

No new data were created or analyzed in this study. Data sharing is not applicable.

## Abbreviations

The following abbreviations are used in this manuscript:

GBM	Glioblastoma
EV	Extracellular Vesicle
MRI	Magnetic Resonance Imaging
CSF	Cerebrospinal Fluid
CNS	Central Nervous System
BBB	Blood–Brain Barrier
FUS	Focused Ultrasound
DEP	Dielectrophoretic
iNTA	Interferometric Nanoparticle Tracking Analysis
HA	Hyaluronic Acid
